# Association of Herpes Simplex Virus Type-1 With Dementia Outcomes: Longitudinal Retrospective Cohort Study Using Real-World Electronic Health Record Data

**DOI:** 10.2196/83028

**Published:** 2026-09-11

**Authors:** Muhammad Muinul Islam, Randi Foraker, Md Saber Hossain, W David Arnold, Dima Dandachi, Abu Saleh Mohammad Mosa

**Affiliations:** 1 Department of Biomedical Informatics, Biostatistics, and Medical Epidemiology, School of Medicine, University of Missouri, 1 Hospital Drive, Columbia, MO, 65201, United States, 1 573 256 9692; 2 Department of Physical Medicine and Rehabilitation, NextGen Precision Health, University of Missouri, Columbia, MO, United States; 3 Department of Neurology, NextGen Precision Health, University of Missouri, Columbia, MO, United States; 4 Department of Medicine, Division of Infectious Diseases, University of Missouri, Columbia, MO, United States; 5 Department of Biomedical Informatics and Data Science, Heersink School of Medicine, The University of Alabama at Birmingham, Birmingham, AL, United States

**Keywords:** HSV-1, herpes simplex virus, dementia, Alzheimer disease, electronic health records, propensity score, aging

## Abstract

**Background:**

Global dementia cases, currently exceeding 55 million, are projected to triple by 2050. In the absence of disease-modifying therapies, identifying modifiable risk factors is critical. Preclinical studies show that herpes simplex virus type-1 (HSV-1) is neurotropic and can drive amyloid-beta accumulation and tau hyperphosphorylation. Epidemiologic findings remain inconsistent, partly because many studies lack standardized designs for real-world data.

**Objective:**

To quantify the association between clinically coded HSV-1 diagnosis and incident cognitive impairment or dementia among patients receiving care in a health-system electronic health record (EHR) network.

**Methods:**

We assembled a retrospective cohort within an Observational Medical Outcomes Partnership (OMOP)–mapped EHR data lake (2010‐2024), comparing patients with a first HSV-1 diagnosis (n=6274) to those without HSV-1 codes but with parallel baseline criteria (n=379,975). Eligibility required at least 365 days of prior observation and absence of baseline cognitive, HIV, selected neurotropic viral, or recent transplant codes. The outcome combined mild cognitive impairment and Alzheimer disease and related dementias (ADRD). A high-dimensional propensity score (PS) incorporating approximately 17,000 baseline covariates was estimated with regularized logistic regression; 4 strata weights were applied in a Cox model. Sensitivity analyses examined equipoise trimming, doubly adjusted models, fixed 1-, 5-, and 10-year censoring horizons, and a dementia-only outcome subset. Negative-control outcomes (n=271) were prespecified to assess empirical calibration.

**Results:**

The HSV-1 cohort contributed 23,186 person-years (PY) and 469 events; the non-HSV-1 cohort contributed 1,519,827 PY and 24,764 events. Crude incidence was 20.23 (95% CI 18.44‐22.14) per 1000 PY in the HSV-1 cohort and 16.29 (95% CI 16.09‐16.50) in the non-HSV-1 cohort, an absolute difference of 3.94 events per 1000 PY. PS-stratified analysis yielded a hazard ratio (HR) of 1.14 (95% CI 1.03‐1.25; *P*=.007). Estimates were comparable after equipoise trimming (HR 1.12, 95% CI 1.01‐1.25, *P*=.04), doubly adjusted modeling (HR 1.13, 95% CI 1.02‐1.24, *P*=.01), and fixed 5-year (*P*=.008) and 10-year follow-up (*P*=.004) (both HR 1.15). The 1-year censored model showed HR 1.00 (95% CI 0.84‐1.20; *P*=.96). The dementia-only endpoint mirrored the primary analysis (HR 1.14, 95% CI 1.03‐1.25; *P*=.008). No postindex events occurred for any negative-control outcome, so empirical calibration could not be performed and all estimates are uncalibrated.

**Conclusions:**

Within an OMOP-standardized EHR cohort, an HSV-1 diagnosis was associated with a small elevation in the hazard of subsequent cognitive impairment or dementia compared with individuals with no recorded HSV-1 diagnosis. Given the ubiquity of HSV-1, even a modest elevation in individual hazard could translate to a meaningful population-level increment if the association is causal. Confirmation in external data sets with laboratory viral typing, antiviral treatment records, and mortality linkage is warranted.

## Introduction

Dementia represents a major public health challenge, with prevalence trends exceeding current therapeutic capacities. Current estimates indicate 55 million individuals live with dementia worldwide, with this figure expected to nearly triple by 2050 as populations age [1-4]. In the United States alone, approximately 7 million adults aged 65 years or older are diagnosed with Alzheimer disease and related dementias (ADRD) [5-8], a number forecast to nearly double by midcentury without effective interventions. While several factors are strongly implicated, the full causal pathway and the degree to which these factors interact over time remain active areas of investigation. In addition, because disease-modifying therapies remain elusive, elucidating modifiable risk factors is essential for shaping effective prevention strategies.

Herpes simplex virus type-1 (HSV-1), a neurotropic virus, establishes lifelong latency in peripheral sensory ganglia and reactivates periodically throughout adulthood [2,5,9-23]. Global seroprevalence estimates indicate widespread exposure, with antibody positivity rates exceeding 65% among individuals older than 50 years [16]. Experimental studies demonstrate that HSV-1 can penetrate the blood-brain barrier, induce microglial activation, and promote amyloid-beta aggregation and tau hyperphosphorylation, which are central features of Alzheimer disease (AD)–related neuropathology [9,19,24-39]. Experimental findings further suggest that recurrent HSV-1 reactivation may trigger or accelerate neuropathological mechanisms underlying mild cognitive impairment and AD, though these relationships have not been directly tested in human epidemiologic studies [30,40-43].

Epidemiological evidence on the relationship between HSV-1 and cognitive outcomes has been mixed [44-54]. Early serological and smaller cohort studies yielded inconsistent associations [42,55,56], possibly due to variations in exposure definitions, outcome ascertainment, and insufficient control for confounders. Large-scale administrative database studies have yielded conflicting results despite improved statistical precision, potentially attributable to variability in antiviral treatment patterns, diagnostic coding practices, and challenges distinguishing between HSV-1 and HSV-2 infections [40,41,55,57-62]. Recent methodological appraisals highlight persistent limitations in the existing literature, emphasizing the need for large-scale, well-controlled longitudinal studies [5,12,15]. A systematic review focusing specifically on the association between HSV-1 and dementia has been registered and its protocol published (PROSPERO CRD42024516789) [12]; however, the completed review had not appeared in the indexed literature at the time of this writing, and well-designed individual studies using structured clinical data with explicit confounding control continue to constitute the primary evidence base.

To address these gaps, we conducted a retrospective cohort study using Electronic Health Record (EHR) data standardized to the Observational Medical Outcomes Partnership (OMOP) Common Data Model (CDM) to evaluate whether a clinically documented HSV-1 diagnosis is associated with an increased hazard of incident cognitive impairment or dementia. The analysis used a new-user (incident-exposure) cohort design with an encounter-anchored comparator and up to 15 years of longitudinal follow-up, high-dimensional propensity score (PS) stratification incorporating approximately 17,000 baseline covariates spanning medication exposures, comorbidity profiles, health care usage patterns, and demographic characteristics, and a prespecified program of sensitivity analyses [12,14]. The present analysis is designed to address several of the methodological gaps anticipated by the systematic review protocol noted above: it uses formal new-user cohort construction with symmetric eligibility and an encounter-anchored comparator, applies high-dimensional PS adjustment, uses an OMOP-standardized phenotype for both exposure and outcome, and isolates HSV-1 using type-specific diagnostic concept sets, features that were absent from most prior EHR-based studies of this association. We hypothesized that coded HSV-1 exposure would be associated with an elevated hazard of cognitive impairment or dementia, consistent with mechanisms proposed in prior laboratory and translational research.

## Methods

### Data Source

We analyzed data from the University of Missouri Health Care Research Data Lake, a repository encompassing approximately 2.2 million patients with clinical encounters recorded between January 1, 2010, and December 31, 2024. The Data Lake integrates structured inpatient, outpatient, emergency department, and specialty clinic records standardized to the OMOP Common Data Model (CDM) version 5.0, enabling consistent phenotype ascertainment across heterogeneous clinical settings [63]. Cohort definitions were implemented in ATLAS version 2.12 (Observational Health Data Sciences and Informatics [OHDSI]) [64,65] and executed in R version 4.4.1 (R Foundation for Statistical Computing) using the OHDSI packages CohortMethod version 4.2.3 [66,67], FeatureExtraction version 3.7.2 [68], and EmpiricalCalibration version 3.1.3 [69,70], together with supporting dependencies. The analytic package (ATLAS JSON cohort definitions, concept sets, executable R scripts, model objects) is archived in a version-controlled public repository.

### Study Design

We conducted a retrospective cohort study to evaluate whether a recorded HSV-1 diagnosis is associated with subsequent incident cognitive impairment or dementia. We used a new-user, incident-exposure cohort design in which both cohorts entered at incident eligibility, had a clean baseline period, and followed parallel exclusion rules. We do not describe this as an active-comparator design because, in pharmacoepidemiology, an active comparator typically includes new users of alternative treatments for the same indication, helping control confounding by indication and healthy-user or frailty bias at the design stage [71]. Comparator entry was anchored to a health-system encounter, defined by an observation period and any recorded condition occurrence. This ensured that comparator members were health-system users with measurable baseline covariates and a defined time zero, rather than untreated general-population members. Thus, the design aligned data provenance, eligibility, and health-system engagement across groups, while control of confounding by indication and related host factors relied on the high-dimensional PS approach, as discussed in the Limitations section. Eligible observation time spanned 2010-01-01 through 2024-12-31. Each participant was required to have at least 365 days of continuous observation prior to cohort entry (baseline wash-in). Follow-up for outcome assessment began 1 day after the cohort index date and continued until the earliest of (1) qualifying outcome, (2) end of continuous observation in the Data Lake, or (3) 2024-12-31 (administrative study end). This approach ensured that follow-up periods were standardized between groups, independent of subsequent diagnostic activity. A schematic of the cohort construction and time-at-risk is provided in Multimedia Appendix 1 (Figure A: analytic workflow diagram and Figure B: cohort logic flow diagram).

### Cohort Definitions

#### HSV-1 Exposure Cohort

The HSV-1 exposure cohort was defined as individuals with a first recorded diagnosis of HSV-1 during the study period (January 1, 2010, through December 31, 2024). Exposure was operationalized strictly on the basis of diagnostic codes, not downstream sequelae. This operationalization therefore identifies clinically coded HSV-1 disease rather than true biological infection status. A coded HSV-1 diagnosis most plausibly reflects a subset of infected individuals whose disease was symptomatic, recurrent, severe enough to prompt medical attention, or clinically recognized by a provider, and who received a corresponding diagnostic code during the encounter. It does not capture asymptomatic primary infection or clinically silent reactivation, which accounts for the majority of HSV-1 carriage in the general population. Given the high background seroprevalence of HSV-1 in the general adult population, the non-HSV-1 comparator therefore represents an absence of coded HSV-1 rather than a confirmed absence of infection and likely includes a substantial proportion of latently infected but never-coded individuals. This nondifferential exposure misclassification is expected to bias effect estimates toward the null and is discussed further in the Limitations section. Codes were drawn from a curated OMOP concept set including *ICD-10-CM* B00.x (herpesviral infections), *ICD-10-CM* A60.x (anogenital herpes attributed to HSV-1), and *ICD-9-CM* 054 .x, and were restricted to HSV-1 specific descendants or mapped concepts; HSV-2 specific and nontyped herpes simplex codes were not used for exposure qualification. A full listing of included concepts is provided in Multimedia Appendix 2. To capture incident HSV-1 exposure and minimize confounding, hierarchical exclusion criteria were applied as detailed below.

Participants were excluded if they had a recorded diagnosis of HIV at any time prior to or on the index date. In addition, individuals with documented infection by other neurotropic viruses including varicella zoster virus, cytomegalovirus, Epstein-Barr virus, or human herpesvirus 6 or 7 within the 365-day baseline period were excluded. The cohort also excluded patients with a history of solid-organ or tissue transplantation within the 90 days preceding the index date, to avoid inclusion of immunosuppressed individuals with elevated risk for neurological complications. To ensure outcome temporality, individuals with any cognitive impairment diagnosis within the 365-day baseline window were excluded. Only the earliest qualifying HSV-1 diagnosis per person was retained for cohort entry. When multiple qualifying records occurred on the same date, they were merged into a single episode using an era-based approach *(EraPad =0 day)*. Because systemic immunomodulatory therapies (eg, high-dose systemic corticosteroids, calcineurin inhibitors, antimetabolites, biologic DMARDs, or cytotoxic chemotherapy) can modify HSV-1 reactivation risk, we did not exclude such patients a priori; instead, baseline medication exposures including immunomodulators were captured in the high-dimensional covariate set and controlled via the PS model (Multimedia Appendix 3). Follow-up began one day after the index event (postindex day 1) and continued until the end of continuous observation.

#### Non-HSV-1 (No Recorded HSV-1 Diagnosis) Comparator Cohort

The non-HSV-1 comparator cohort was drawn from the same Data Lake and calendar frame. Cohort entry events were generated from observation periods within the study dates combined with any recorded condition occurrence to ensure an encounter-anchored index (full listing in Multimedia Appendix 4). The earliest qualifying event per person was used. Inclusion required: (1) no recorded HSV-1 diagnosis at any time prior to index; (2) no HIV infection at or before index; (3) no specified other neurotropic viral infection from 365 days before through index; (4) no solid-organ or tissue transplant from 90 days before through index; and (5) no cognitive-impairment codes in the 365-day baseline. Follow-up and exit rules matched the exposure cohort (end of continuous observation). Because comparator entry was anchored to a health-system encounter rather than an HSV-1 diagnosis, the comparator cohort shared data provenance, baseline eligibility, and health-system engagement with the HSV-1 cohort, but not a common clinical indication. We therefore characterize it as an encounter-anchored, no-recorded-HSV-1 comparator rather than an active comparator; the implications for residual confounding are addressed in the Limitations section.

### Outcome Definition

The primary endpoint was incident all-cause cognitive impairment or dementia, ascertained using a curated OMOP concept set of more than 120 *ICD-9-CM* and *ICD-10-CM* codes spanning mild cognitive impairment, ADRD (vascular, frontotemporal, Lewy body), amnestic syndromes, and nonspecific cognitive deficit codes; the full listing is provided in Multimedia Appendix 5. Individuals with any code from this set during the 365-day baseline were excluded from both cohorts. The earliest qualifying postindex diagnosis defined the outcome event; both inpatient and outpatient settings were permitted.

### Covariates

"Covariates for PS modeling were identified using a high-dimensional feature extraction process implemented through the FeatureExtraction package version 3.7.2 (Observational Health Data Sciences and Informatics [OHDSI], coordinating center at Columbia University, New York, NY, United States) [68].". Approximately 17,000 candidate baseline covariates were considered, capturing clinical features from two distinct observational windows prior to the index date: a long-term period (day −365 through day −31) and a short-term period immediately preceding cohort entry (day −30 through day −1). These covariates represented 5 broad domains, including medication use, clinical procedures, condition history, aggregated clinical measurements, and indicators of health care usage. In addition, patient demographic characteristics encompassing age categories, sex, race, ethnicity, and calendar timing (index month and year) were explicitly included. To prevent redundancy within the large covariate set, intermediate clinical measurement windows and standardized composite indices such as *Charlson* and *CHADS2* scores were deliberately excluded. Rare covariates were retained without imposing frequency thresholds, and regularized logistic regression was utilized to handle sparse data. No imputation was performed for missing values; race or ethnicity data classified as “unknown” or “not recorded” in the source records were retained as distinct categories. Continuous covariates were converted to categorical or binary indicator variables per default *FeatureExtraction* processing methods.

### Propensity Score Estimation

A PS for HSV-1 exposure was estimated using regularized logistic regression implemented in *CohortMethod* with a Laplace (L1) prior with variance 1 and tuned by ten-fold cross-validation. All 17,154 candidate baseline covariates were used in the model; 826 retained nonzero coefficients after shrinkage. Coefficients ranged from −6.56 to +2.62 (median −0.006; IQR −0.12 to +0.11). The full coefficient vector with OMOP covariate identifiers is available in Multimedia Appendix 6 to permit external reconstruction of the PS.

### PS Implementation and Balance Assessment

**Figure 1.**
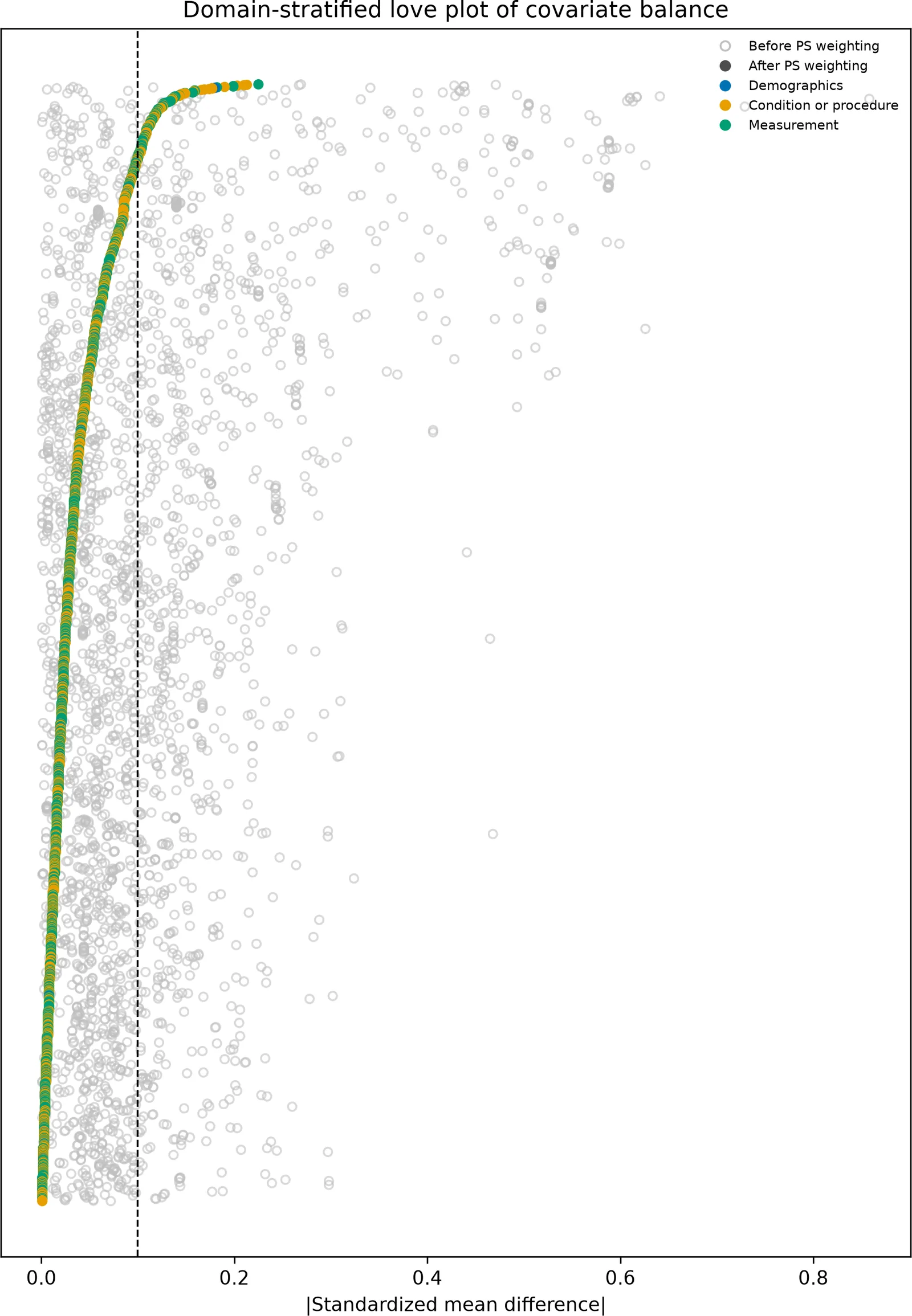
Domain-stratified covariate balance plot (Love plot) before and after PS weighting. PS: propensity score.

**Figure 2.**
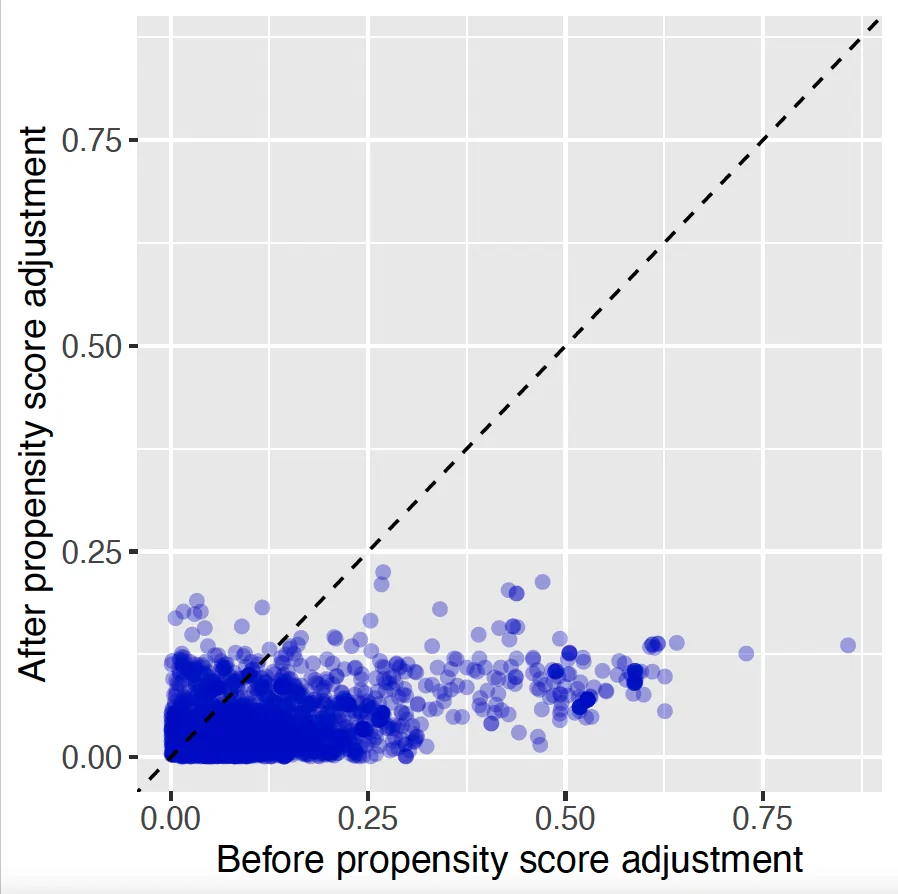
Covariate balance before versus after propensity score weighting. Each point represents one baseline covariate (n≈17,000). The dashed 45° line marks no change; points below the line indicate improved balance. Postweighting, >99 % of covariates lie below |SMD|=0.10, confirming that the propensity-score procedure effectively eliminated measurable baseline differences between cohorts.

Individuals were divided into four PS strata using *CohortMethod* defaults (strata defined to contain equal numbers of target persons). Stratum weights were applied in downstream effect estimation; no trimming was used in the primary analysis. Baseline balance was evaluated using the *CohortMethod* and *covariateBalance* output. For each of the 17,154 covariates we computed prevalence (or mean for continuous measures) by cohort and the absolute standardized mean difference (|SMD|) before and after PS stratification. Residual imbalance was considered acceptable if |SMD| was less than 0.10. Summary diagnostics appear in Figure 1 (domain-stratified Love plot) and Figure 2 (covariate-level balance scatterplot).

### Attrition and Analytic Sample

**Figure 3.**
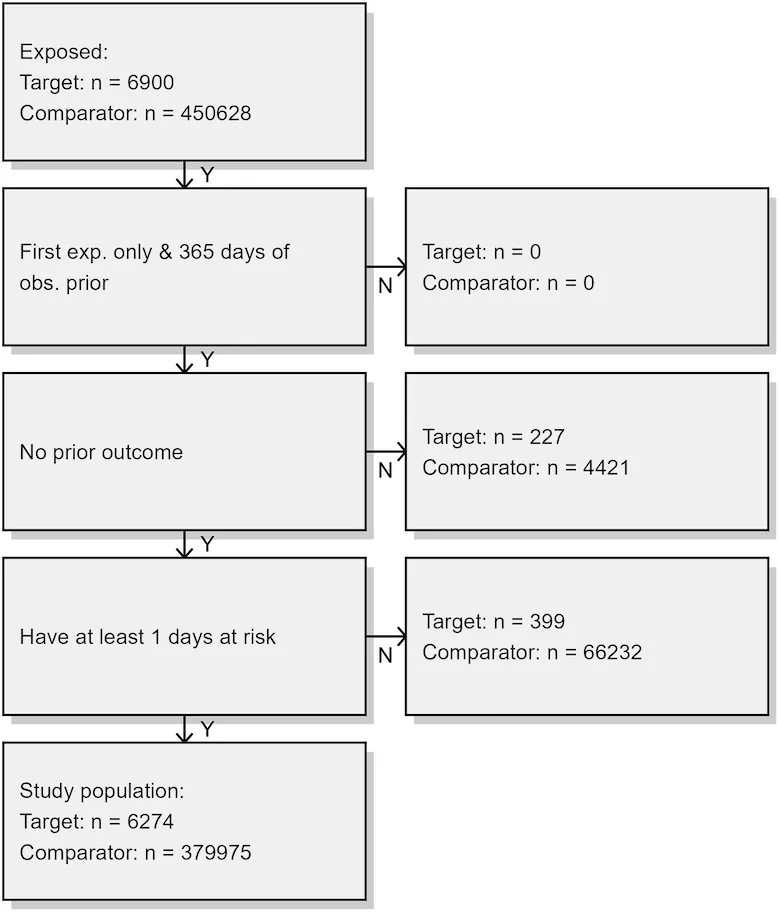
Cohort attrition flow diagram. Sequential application of eligibility and exclusion criteria to derive the HSV-1 and non-HSV-1 cohorts from the University of Missouri Research Data Lake (2010‐2024). Numbers at right show exclusions at each step; final analytic counts were 6274 HSV-1 and 379,975 non-HSV-1. For further details related to HSV-1, see Table 1. HSV: herpes simplex virus type-1.

Eligibility was applied sequentially using the criteria described above. The composition of the resulting analytic cohorts is summarized in Table 1 (attrition table) and illustrated in Figure 3 (cohort flow diagram). Person-time and outcome counts are reported in the Results section.

**Table 1.** Cohort attrition from candidate pools to analytic sample.

Exclusion step	HSV-1 ^a^ candidates remaining	HSV-1 removed at step	Comparator candidates remaining	Comparator removed at step	Step attrition % (HSV-1)	Step attrition % (Comp)	Cumulative attrition % (HSV-1)	Cumulative attrition % (Comp)
Candidate population	6900	—^b^	450,628	—	—	—	—	—
First qualifying event and ≥365 days prior observation	6900	0	450,628	0	0	0	0	0
No prior outcome (365-days washout)	6673	227	446,207	4421	3.3	1	3.3	1
≥1 day at risk postindex	6274	399	379,975	66,232	6	14.8	9.1	15.7
Final analytic cohort	6274	—	379,975	—	—	—	9.1	15.7

a HSV: herpes simplex virus type-1.

b Not available.

### Primary Effect Estimation

The primary association between HSV-1 diagnosis and incident cognitive impairment or dementia was estimated using a Cox proportional hazards model stratified by PS stratum. Time zero was index date plus 1 day. Individuals were followed until outcome, end of continuous observation, or 31 December 2024, whichever occurred first. Hazard ratios with 95% CIs are reported in the Results section.

### Negative-Control Outcomes and Calibration

We prespecified 271 negative-control outcomes (conditions with no plausible causal relationship to HSV-1, eg, psoriasis, osteoarthritis; complete list in Multimedia Appendix 7) to assess residual systematic error. Each control outcome was analyzed through the same PS-stratified Cox workflow used for the primary endpoint. Empirical null distributions were to be estimated from these controls to calibrate effect estimates following the approach of Schuemie et al [69,70]; the results of this calibration step are reported in the Results section.

### Statistical Power

Minimum detectable relative risk was calculated using *CohortMethod* power diagnostics, assuming 80% power and two-sided α=.05. The calculation was applied to observed person-time and event counts from the PS-adjusted analytic sample; these values are reported in the Results section in Table 2.

**Table 2.** Cohort person-time, events, crude incidence rates, and minimum detectable relative risk.

Metric	HSV-1^a^ (Target)	Non-HSV-1^a^ (Comparator)
Subjects	6274	379,975
PY^b^	23,186	1,519,827
Events	469	24,764
Incidence rate/1000 PY^b^	20.23	16.29
Minimum detectable RR^c^ (80% power)	—^d^	1.15

a HSV-1: herpes simplex virus type-1.

b PY: person-years

c RR: relative risk.

d Not applicable.

### Software and Reproducibility

Analyses were conducted in R version 4.4.1 (R Foundation for Statistical Computing) using the open-source packages CohortMethod version 4.2.3 [66,67], FeatureExtraction version 3.7.2 [68], and EmpiricalCalibration version 3.1.3 [69,70], all developed and maintained by Observational Health Data Sciences and Informatics (OHDSI), together with related dependencies. All ATLAS (developed by OHDSI) JSON cohort definitions, concept set files, analysis settings, and executable code required to reproduce the results are available in a public GitHub (Microsoft Corporation) repository [72].

### Ethical Considerations and Data Governance

This secondary analysis of deidentified University of Missouri (MU) Health system EHR data was reviewed by the University of Missouri Institutional Review Board and determined to be non-human subjects research (IRB #2114987 MU); informed consent was waived. Analyses were conducted within a secure University of Missouri analytic environment compliant with (Health Insurance Portability and Accountability Act) HIPAA and institutional data governance policies. This study adheres to the (Strengthening the Reporting of Observational Studies in Epidemiology) STROBE guidelines; the completed checklist is provided in Checklist 1.

## Results

The following results refer to the analytic cohorts assembled as described in the Methods section. Unless otherwise specified, counts and person-time are unweighted, and “HSV-1 cohort” refers throughout to individuals meeting the type-specific diagnostic code definition described above.

### Cohort Accrual

We identified 6900 candidate HSV-1 index events and 450,628 potential comparator candidates in the University of Missouri Research Data Lake for the 2010‐2024 study window. After applying the continuous prior observation requirement (at least 365 days), exclusion criteria (HIV, specified other neurotropic infections, transplant), and the 365-day baseline washout for prior cognitive diagnoses, the analytic cohorts included 6,274 HSV-1 and 379,975 non-HSV-1 (no recorded HSV-1 diagnosis) individuals (about 60:1 comparator to HSV-1). Baseline attrition was 9.1% in the HSV-1 cohort and 15.7% in the comparator cohort.

### Follow-Up Time

The distribution of follow-up times was right-skewed in both cohorts, reflecting a minority of individuals with extended observation periods. Median follow-up was 1111 days (IQR 413‐2,631; 90th percentile 2924; maximum 5460) in HSV-1 participants and 956 days (interquartile range 327‐3165; 90th percentile 3708; maximum 5478) in non-HSV-1 participants (Table 3). These distributions informed the prespecified administrative censoring horizons evaluated in sensitivity analyses (1 year, 5 years, and 10 years).

**Table 3.** Distribution of individual follow-up time (days) by cohort.

Cohort	Min	P10	P25	Median	P75	P90	Max
HSV-1(Target)^a^	1	140	413	1111	2631	2924	5460
Non-HSV-1 (Comparator)	1	71	327	956	3195	3706	5476

a HSV: herpes simplex virus type-1.

### Person-Time, Events, and Statistical Power

Among participants retained in the analytic sample, the HSV-1 cohort contributed 23,186 PY and 469 incident all-cause cognitive impairment events; the non-HSV-1 cohort contributed 1,519,827 person-years and 24,764 events. Given these observed rates and sample sizes, the study achieved 80% power to detect HRs of 1.15 or greater (two-sided α=.05). Crude incidence rates are shown in Table 2.

### Crude Incidence Rates of All-Cause Cognitive Impairment

Unweighted crude incidence of the primary outcome was 20.23 per 1000 PY (95% CI 18.44‐22.14) in HSV-1 and 16.29 per 1000 person-years (95% CI 16.09‐16.50) in non-HSV-1 participants. The absolute rate difference was about 3.94 events per 1000 PY (one additional event for every approximately 254 PY of follow-up among HSV-1 participants compared with non-HSV-1). These crude findings were consistent in direction with the adjusted HR.

### Baseline Characteristics and PS Balance

Before PS adjustment, the HSV-1 cohort had a higher proportion assigned female at birth (Table 4) and a lower proportion aged 0‐4 years than the comparator cohort, with 72.1% (4976/6900) vs 52.8% (132,122/250,000) female and 2.7% (189/6900) vs 11.3% (28,370/250,000) aged 0‐4 years. Four-stratum PS weighting reduced these imbalances. With an absolute |SMD| threshold of less than 0.10, all demographics displayed in Table 4 met balance after weighting except age 0‐4 years (|SMD| 0.18) and race (Black |SMD| 0.11; White |SMD| −0.13). These residual differences were addressed in a doubly robust sensitivity model. Global balance across approximately 17,000 covariates is shown in the domain-stratified balance plot (Figure 1), and nearly all postweighting covariates had |SMD| less than 0.10. Baseline characteristics before and after weighting are summarized in Table 4, and full covariate-level results are provided in Multimedia Appendix 3.

**Figure 4.**
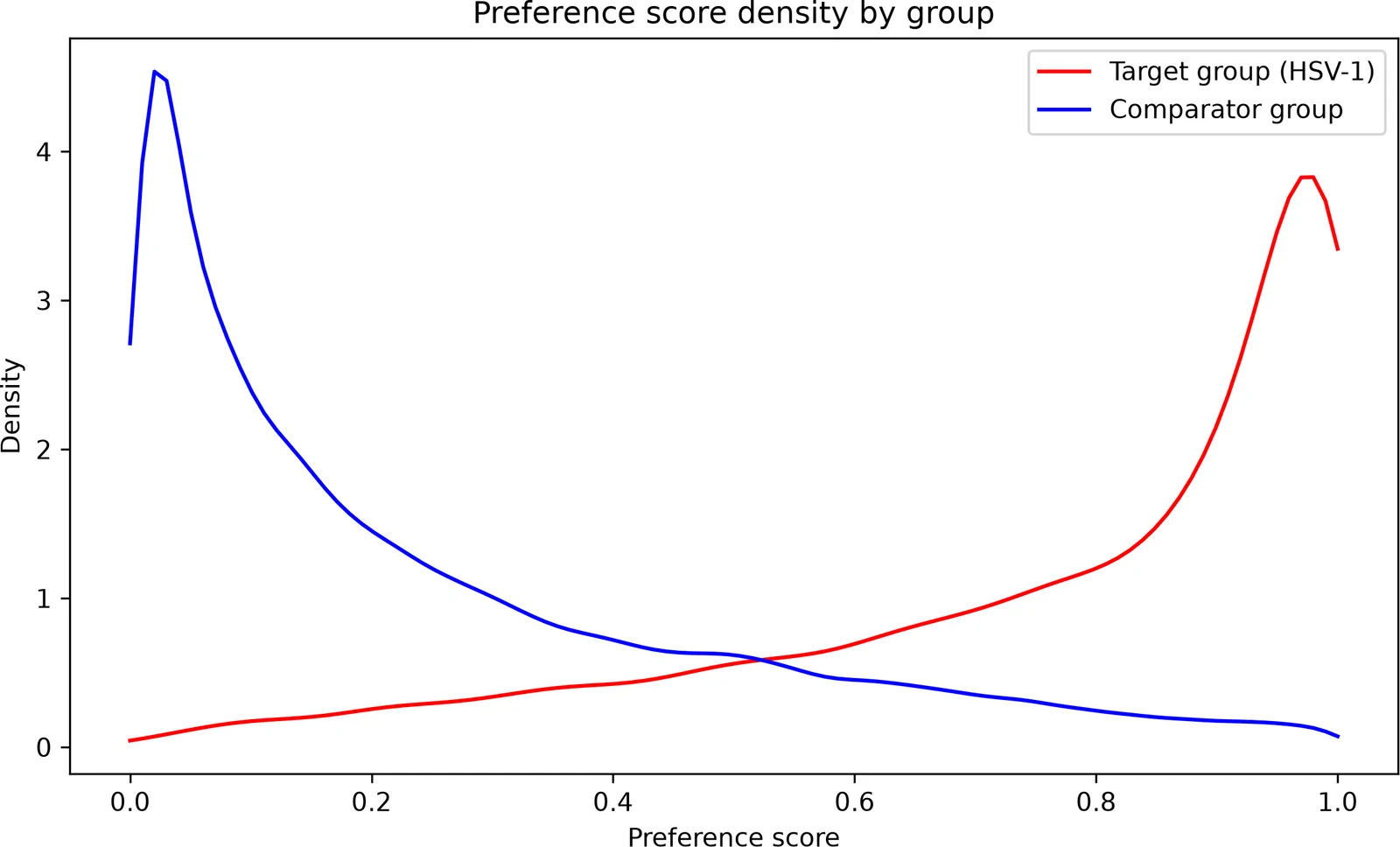
Kernel-smoothed preference score distributions for HSV-1 (red) and non-HSV-1 (blue) cohorts. HSV: herpes simplex virus type-1.

Figures 1 and 4 depict the preference score distribution & domain-stratified covariate balance plot.

Empirical overlap in the preference score distribution *(min{PS, 1 - PS})* was visualized with density plots; trimming thresholds used in sensitivity analyses are marked at 0.10 and 0.90.

**Table 4.** Baseline characteristics before and after propensity score (PS) weighting.

Characteristic	Before target	Before comp	Before SMD^a^	After target	After comp	After SMD
Age 0‐4, years	2.7	11.3	−0.34	18.4	12	0.18
Age 5‐9, years	2.8	5.6	−0.14	7.8	5.5	0.09
Age 10‐14, years	2.2	4.8	−0.14	5.2	4.7	0.02
Age 15‐19, years	4.8	6.2	−0.06	6.8	6.3	0.02
Age 20‐24, years	12.1	15.4	−0.1	12.5	14.8	−0.07
Age 25‐29, years	8.6	6.2	0.09	4.8	6.1	−0.06
Age 30‐34, years	8.5	5.5	0.12	4	5.4	−0.07
Age 35‐39, years	8.1	4.7	0.14	4.3	4.7	−0.02
Age 45‐49, years	6.7	4.4	0.1	4.5	4.5	0
Age 50‐54, years	6.7	4.7	0.09	3.3	4.7	−0.07
Age 55‐59, years	6.2	4.8	0.06	3.1	4.9	−0.09
Age 60‐64, years	6.9	4.9	0.09	4.8	4.9	−0.02
Age 65‐69, years	6	4.4	0.07	4	4.4	−0.02
Age 70‐74, years	4.5	3.5	0.05	3	3.4	−0.03
Age 75‐79, years	3.2	4.5−	−0.07	4.6	4.5	0
Age 80‐84, years	1.7	3.1	−0.09	3.1	3	0
Age 85‐89, years	1.1	1.8	−0.06	1.5	1.7	−0.02
Gender: women	72.1	52.8	0.41	51.9	53.9	−0.04
Race: Asian	1.8	2.2	−0.03	3.7	2.2	0.09
Race: Black or African American	8.9	7.1	0.07	10.2	7.2	0.11
Race: White	85.2	84.7	0.01	80.3	85.1	−0.13
Race: Native Hawaiian or other Pacific Islander	0	0.1	−0.01	0.1	0.1	0
Race: refuse to answer	0.3	0.6	−0.04	0.7	0.6	0.01
Ethnicity: Hispanic or Latino	2.9	2.9	0	3.5	3	0.03
Ethnicity: not Hispanic or Latino	96.4	91.2	0.22	90.5	92.3	−0.06

a SMD: standardized mean difference.

### PS Diagnostics

**Figure 5.**
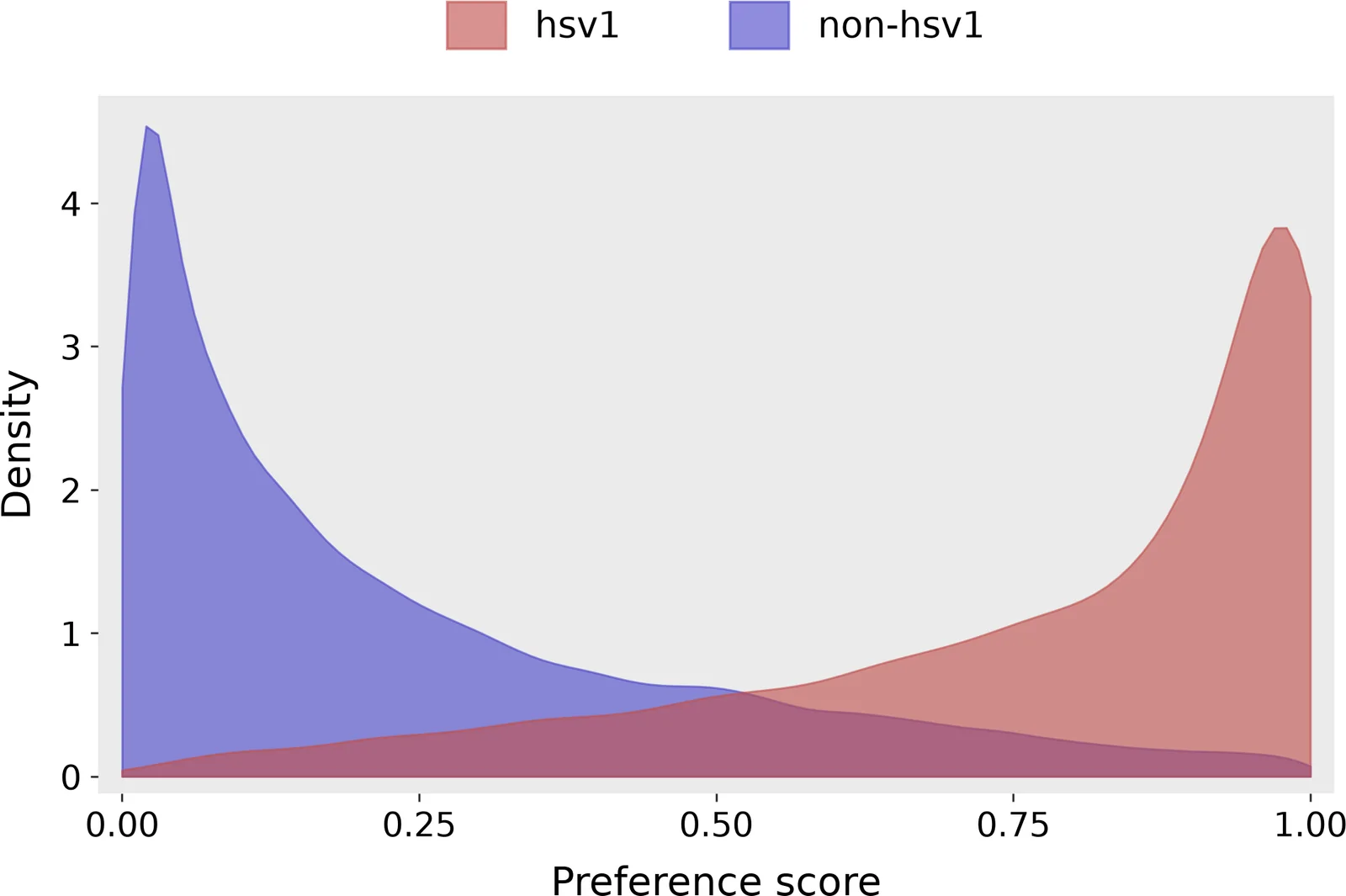
Preference score density distributions by cohort. HSV: herpes simplex virus type-1.

PS density plots (Figures 4 and 5) showed a high concentration of HSV-1 participants at higher exposure probabilities and non-HSV-1 participants at lower probabilities, with limited overlap between about 0.30 and 0.70, consistent with strong model discrimination *(c-statistic approximately 0.92)*. The full distribution of preference scores in exposure and comparator cohorts is provided in Multimedia Appendix 8. However, trimming observations with preference scores below 0.10 or above 0.90 retained 89 % (5584/6274) of HSV-1 and 84 % (319,179/379,975) of non-HSV-1 participants and was evaluated as a sensitivity analysis.

Figures 2 and 5 show preference score density distributions by cohort and covariate balance scatterplot before vs after weighting.

### Primary Effect Estimate: PS-Stratified HR

In the PS-stratified Cox proportional hazards model, HSV-1 diagnosis was associated with a 14% higher hazard of incident all-cause cognitive impairment (HR 1.14; 95% CI 1.03‐1.25; *P*=.007).

**Figure 6.**
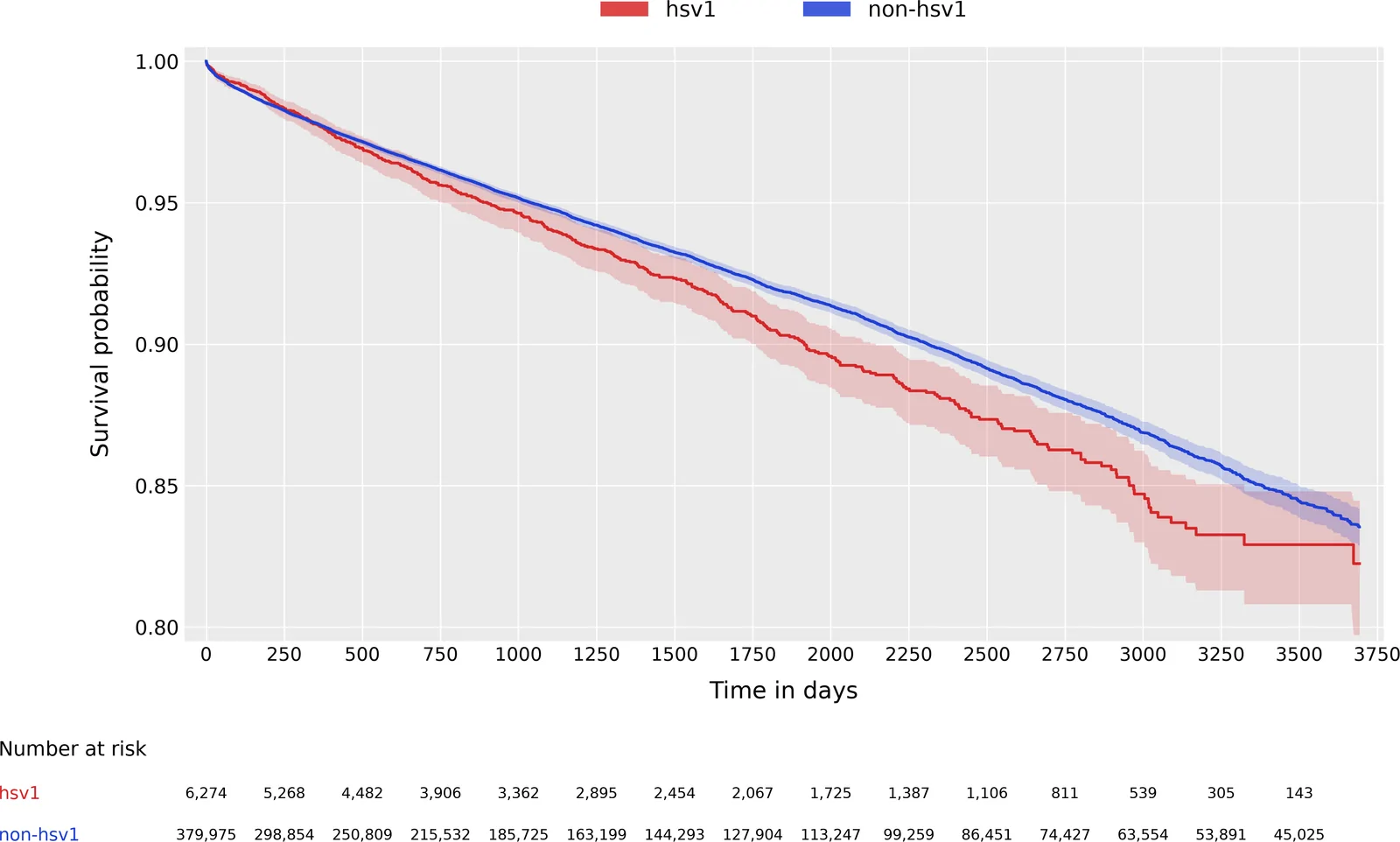
Kaplan-Meier curves for remaining free of all-cause cognitive impairment up to 10 years, weighted by propensity score (PS) strata; ribbons show 95 % CI. HSV: herpes simplex virus type-1

**Figure 7.**
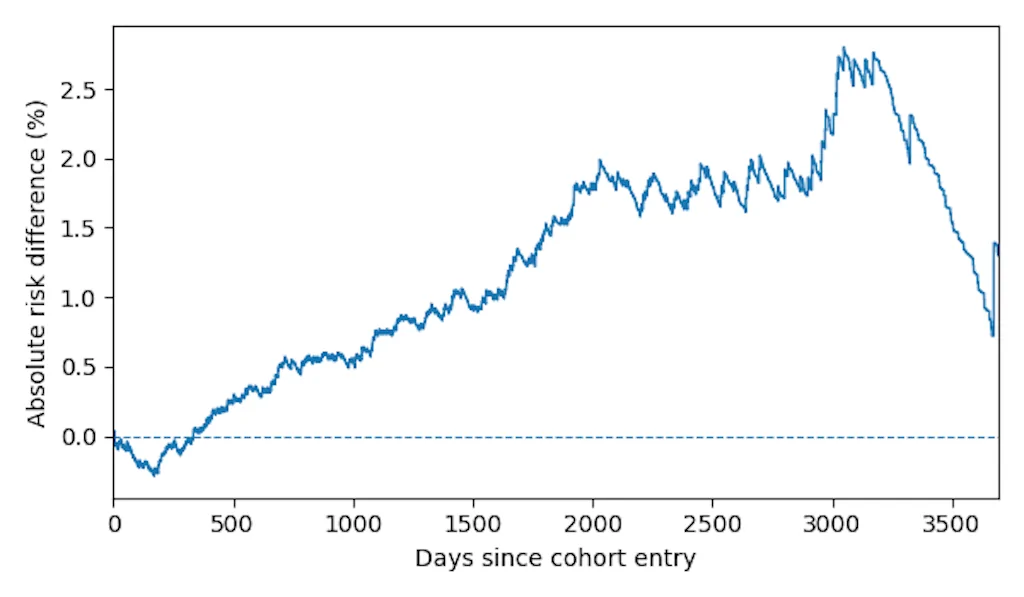
Cumulative absolute risk difference (non-HSV-1 minus HSV-1) over 10 years. Day-by-day absolute difference in survival probability. Positive values indicate excess risk in HSV-1. HSV: herpes simplex virus type-1.

**Figure 8.**
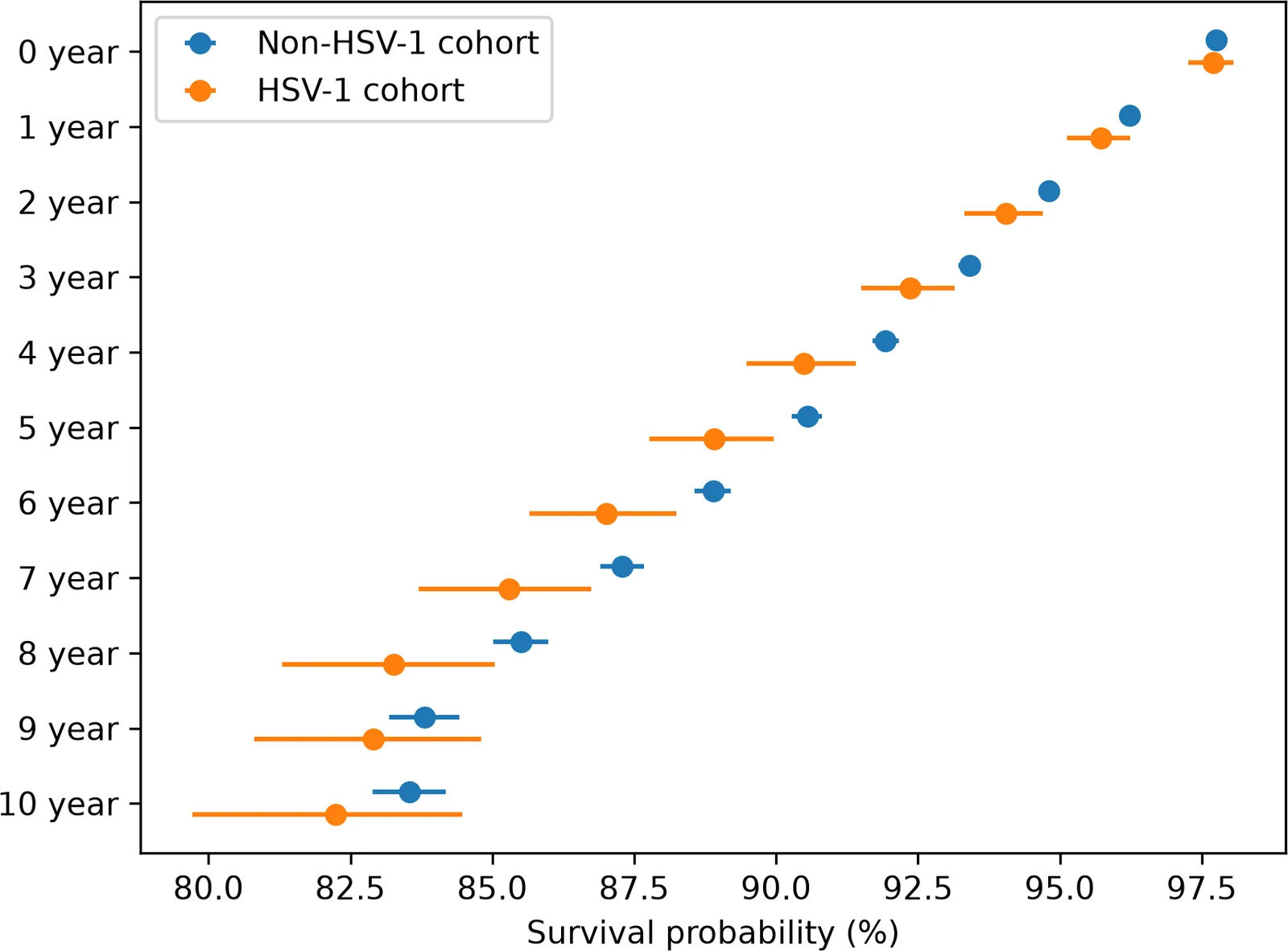
Annual Kaplan-Meier survival probabilities with 95% CI for HSV-1 (orange) and non-HSV-1 (blue) cohorts at years 1‐10 postindex. HSV: herpes simplex virus type-1.

Figures 6–8 show Kaplan-Meier survival curves, cumulative absolute risk difference, and annual survival probabilities.

### Cumulative Incidence Over Time

Kaplan-Meier curves weighted by PS strata showed gradual divergence over follow-up, with clearer separation emerging over extended follow-up (Figure 6 for KM curves; Figure 7 for absolute risk difference; Figure 8 for annual survival probabilities). At 1-year postindex, cognitive disorder-free survival was about 97.7% in HSV-1 and 97.8% in non-HSV-1 participants, a difference of less than 0.1 percentage points. The gap widened to about 2.3 percentage points near year 8 and narrowed in late follow-up (about 1‐2 percentage points by year 10 (Figure 8); wider confidence limits because of attrition). The cumulative absolute risk difference curve (non-HSV-1 minus HSV-1) and annual survival probability plot illustrate these patterns.

The absolute risk difference curve (Figure 7) increased steadily from cohort entry to a peak of approximately 2.8 percentage points near day 3048 (approximately 8.3 years of follow-up), then declined in the final observational period. This pattern reflects primarily the depletion of the HSV-1 at-risk pool in the tail rather than a true convergence of cumulative risk. At day 3000, 539 HSV-1 participants remained at risk compared with 63,554 comparator participants; by day 3500, these counts were 143 and 45,025 respectively, a proportional depletion approximately 2.5 times greater in the HSV-1 group. This asymmetry arises because most HSV-1 individuals entered the cohort after 2010, anchored on their incident diagnosis, and the 2024 administrative end date therefore limits maximum observable follow-up disproportionately for those enrolled later. With fewer than 150 HSV-1 participants at risk beyond day 3500, the Kaplan-Meier estimator becomes unstable: individual outcome events produce large step-function changes in the HSV-1 survival estimate, and the derived absolute difference fluctuates accordingly. Additionally, individuals still event-free at more than 8 years represent a highly selected survivor population, which may attenuate the apparent relative difference independent of statistical noise. For these reasons, point estimates of the absolute risk difference beyond year 8 should be interpreted with caution; the 5- and 10-year administratively censored sensitivity analyses (Table 5) provide more stable comparisons over those defined horizons.

**Table 5.** Summary of sensitivity analyses (preference score trimming, doubly robust model, administrative censoring, dementia-only endpoint).

Analysis	Horizon	HR^a^	95% CI	*P* value^b^	Notes
Preference-score trimming	<0.10 or>0.90 removed	1.12	1.01‐1.25	.04	Sensitivity
Doubly robust (add age 0‐4 years, race White, race Black)	—^c^	1.13	1.02‐1.24	.01	Sensitivity
Admin censor 1 year	Postindex	1.00	0.84‐1.20	>.99	Reconstructed PS-strat^d^ Cox
Admin censor 5 years	Postindex	1.15	1.04‐1.28	.008	Reconstructed PS-strat Cox
Admin censor 10 years	Postindex	1.15	1.04‐1.26	.004	Reconstructed PS-strat Cox
Dementia-only outcome	ADRD^e^ subset	1.14	1.03‐1.25	.008	Sensitivity

a HR: hazard ratio.

b Note: Two-sided *P* value computed from HR and 95% CI as described above; values are rounded to three significant digits.

c Not available.

d PS: propensity score.

e ADRD: Alzheimer disease and related dementias.

### Negative-Control Outcomes

In the MU Data Lake extract, no postindex events were observed in either cohort for any of the 271 prespecified negative-control outcomes (0 events across all 271 outcomes in both the HSV-1 [n=6274] and non-HSV-1 [n=379,975] groups; full listing in Multimedia Appendix 7). Because no events occurred, an empirical null distribution could not be constructed, and effect calibration was not performed; all reported HRs are therefore uncalibrated. This finding likely reflects the rarity of the selected control conditions within the single-institution study window and the relatively modest size of the HSV-1 cohort, rather than true absence of these conditions in the broader population.

### Sensitivity Analyses

Sensitivity analyses demonstrated consistent findings across multiple analytic strategies. After applying preference-score trimming, excluding participants with preference scores below 0.10 or above 0.90, the adjusted HR was 1.12, (95% CI 1.01‐1.25). In a doubly adjusted model accounting for residual imbalance in the age 0‐4 years and race (White; Black or African American) covariates, the estimated HR was 1.13 (95% CI 1.02‐1.24). Administrative censoring at one-year postindex showed no measurable association (HR 1.00, 95% CI 0.84‐1.20), a finding consistent with the low absolute number of outcome events accruing within the first year of follow-up and indicating that the primary signal emerges over extended follow-up rather than in the immediate postindex period. Censoring at five-year and ten-year horizons yielded HRs consistent with the primary finding (HR 1.15, 95% CI 1.04‐1.28, and HR 1.15, 95% CI 1.04‐1.26, respectively), confirming that the association becomes statistically detectable only as cumulative follow-up time increases. Restricting the outcome definition to dementia attributable to Alzheimer disease produced a HR identical to the primary estimate (HR 1.14, 95% CI 1.03‐1.25), reinforcing the stability of findings across these analytic scenarios.

## Discussion

### Principal Findings

Among 386,249 participants followed for up to 15 years, individuals with a clinically documented HSV-1 diagnosis experienced a 14% higher hazard of incident cognitive impairment or dementia than comparator participants without coded HSV-1 diagnoses (HR 1.14, 95% CI 1.03‐1.25, *P*=.007). Throughout this analysis, “HSV-1 exposure” refers specifically to a coded HSV-1 diagnosis in structured EHR data, which most plausibly reflects symptomatic or clinically recognized disease rather than latent or asymptomatic infection; the clinical phenotype captured is therefore a subset of the larger infected population identifiable only by serology. The absolute incidence was approximately 4 events per 1000 PY higher in the HSV-1 cohort (Table 2). PS-weighted survival curves showed gradual divergence that became progressively more apparent and sustained across extended follow-up (Figures 6–8). This pattern is consistent with the sensitivity analyses, in which the 1-year administratively censored model yielded no measurable association, whereas the 5-year and 10-year censored models produced HRs consistent with the primary estimate, together indicating that the association accumulates over extended follow-up rather than being concentrated in the immediate postindex period. The high seroprevalence of HSV-1 in the general adult population means that, if the observed association reflects a causal relationship, even a 14% increase in individual hazard could translate to a meaningful population-attributable increment in dementia cases, a point elaborated in the implications section below.

### Comparison With Prior Work

Prior epidemiological investigations have reported heterogeneous results, likely reflecting differences in case definitions, study design, and confounding adjustment. A nested case-control study using Swedish registry data reported elevated odds of AD in individuals with prior HSV-1 infection, suggesting a temporal link between infection and diagnosis [61]. A Taiwan-based retrospective cohort study using national claims data found increased dementia incidence among patients with HSV diagnoses, though that study combined HSV-1 and HSV-2 under a single code [17]. Our HR is directionally concordant but smaller, possibly reflecting our narrower HSV-1 concept set, finer confounder adjustment via high-dimensional PS stratification, and the single health-system EHR source [61,73]. A parallel analysis by Chen et al [59] examining antiviral treatment in herpetic infections suggested a protective association, but that study did not isolate HSV-1 and did not use PS methods [73].

The biological plausibility of the observed association is supported by mechanistic evidence. Experimental models show that HSV-1 can induce tau hyperphosphorylation and amyloid-beta aggregation in neuronal cultures [11,74,75], disrupt tau exon splicing in human neuron preparations [76,77], and accelerate microglial activation in murine AD models [78,79]. A prospective study within the Multidomain Alzheimer Preventive Trial cohort detected HSV-1 DNA in peripheral blood mononuclear cells and found seropositivity associated with abnormal phosphorylated tau and Abeta42/40 ratios [2,40,61]. The temporal association between HSV-1 diagnosis and cognitive impairment in our cohort is consistent with, but does not directly test, these mechanistic hypotheses; biomarker and laboratory data that would be required to evaluate them were unavailable in this study.

The magnitude of the observed association in this study (HR 1.14) is smaller than estimates from several earlier investigations, and misclassification of exposure is the most parsimonious explanation for this discrepancy. Studies reporting HRs or odds ratios of 1.5 or greater for HSV and dementia have generally used seroprevalence data or seroconversion designs [25,60], in which the exposed group includes all seropositive individuals regardless of whether they received a clinical diagnosis code. In contrast, EHR-based studies restricted to coded diagnoses, as in the present analysis, identify only a small, clinically visible fraction of the truly exposed population. The non-HSV-1 comparator group therefore includes a large number of latently infected individuals, diluting the between-group difference and attenuating the observed HR toward the null. Rather than indicating that the true association is smaller than previously reported, our estimate is best interpreted as a lower bound of the true association under the assumption of nondifferential misclassification. This interpretation is consistent with the systematic evidence gap that Hong et al [12] identified when publishing their systematic review protocol in 2025, namely that the field lacks adequately powered, methodologically rigorous longitudinal studies using structured clinical data with explicit confounding control, a gap the present analysis is specifically designed to address.

### Implications for Practice and Research

The present findings carry several specific implications for clinical practice, health system design, and future research, even in the absence of causal confirmation. Before describing these, we emphasize that a HR of 1.14, while statistically meaningful and directionally consistent across sensitivity analyses, represents a modest effect at the individual patient level and does not, in isolation, justify any change to current clinical management of HSV-1 infection or to existing cognitive-screening recommendations. The findings are intended to guide future research rather than to inform immediate changes in care. At the clinical level, the results suggest that a coded HSV-1 diagnosis, already routinely captured in structured EHR data, may serve as a readily available indicator for an elevated likelihood of subsequent cognitive impairment that clinicians could factor into decisions about cognitive screening. This does not imply that every patient with a cold sore diagnosis requires neurological surveillance; the absolute incidence difference of approximately 4 events per 1000 PY is modest. However, in older patients with additional factors associated with dementia, including advanced age, apolipoprotein E. (APOE) epsilon4 carrier status, and cardiovascular disease burden, a history of clinically documented HSV-1 infection may justify earlier cognitive screening or enrollment in prevention programs. Health systems with OMOP-standardized data could operationalize such surveillance without additional data collection.

At the population level, the estimated seroprevalence of HSV-1 exceeding 65% among adults over 50 years means that even a HR of 1.14 translates to a nontrivial population-attributable fraction of late-life cognitive impairment. If the association is causal, a meaningful proportion of late-life cognitive decline may be linked to this common, potentially modifiable exposure. Antiviral therapies that suppress HSV-1 reactivation, including acyclovir and valacyclovir, are safe, inexpensive, and widely available. Several observational studies have suggested that treated patients have lower dementia incidence than untreated patients [59,61,80], and a registry-based cohort study reported an association between antiherpetic therapy and reduced AD incidence [61]. These reports are hypothesis-generating and are subject to confounding by indication, healthy-user effects, and adherence-related bias; they do not by themselves establish that antiviral therapy modifies dementia risk. Taken together with the mechanistic and epidemiologic evidence summarized above, our findings support continued investigation into whether HSV-1 reactivation contributes meaningfully to cognitive decline and whether antiviral strategies targeting reactivation warrant formal evaluation in appropriately designed prospective studies. The present analysis does not, on its own, provide a basis for recommending antiviral prophylaxis for dementia prevention.

This study provides an analytical template, including OMOP-standardized cohort definitions, open-source ATLAS and *CohortMethod* code, and a replicable PS framework, that could support replication across multiple OMOP-standardized health systems prior to trial design. Prospective studies that distinguish HSV-1 from HSV-2 serologically, quantify viral reactivation frequency, capture antiviral treatment patterns, and incorporate genetic susceptibility markers, including APOE genotype and markers of immune response such as IGDCC4 [81], are needed to determine whether antiviral therapy or immunomodulation could modify dementia trajectories.

Large, multisite studies integrating laboratory-confirmed infection data, pharmacy claims, and vital status linkage will be necessary to validate these findings and to clarify whether virologic suppression is associated with reduced dementia incidence at the population level.

### Strengths and Limitations

#### Strengths

The methodological design of this study is specifically constructed to address the threats to validity that have limited prior EHR-based investigations of HSV-1 and cognitive outcomes, and each design element corresponds to a known weakness in the existing literature. The new-user design with symmetric eligibility and an encounter-anchored comparator is the design element that most clearly separates this study from prior EHR-based analyses, which have generally used prevalent-user or simple-incidence designs. By requiring both HSV-1 and comparator cohort members to have at least 365 days of prior continuous observation free of the outcome, and by anchoring comparator entry on an encounter-based event rather than arbitrary calendar assignment, the design ensures that baseline covariate measurement, outcome washout, and follow-up rules are symmetric across groups. This approach eliminates immortal time bias and reduces index event confounding, two threats that prevalent-user designs cannot adequately address.

The use of OMOP CDM standardization with type-specific HSV-1 concept sets represents a substantive advance over prior studies that used broad herpesvirus codes or administrative claims codes that cannot distinguish HSV-1 from HSV-2. The curated concept set, restricted to *ICD-10-CM* B00.x, A60.x (HSV-1-attributed anogenital herpes), and *ICD-9-CM* 054 .x descendants, and explicitly excluding nontyped codes, produces the most phenotypically specific coded HSV-1 exposure definition achievable from structured EHR data without laboratory linkage.

The high-dimensional PS, incorporating approximately 17,000 baseline covariates spanning medication use, condition history, procedures, clinical measurements, and health care usage, addresses a limitation that affects smaller studies: residual confounding from unmeasured or partially measured baseline differences. The L1-penalized model ensures parsimony while retaining flexibility, and the full coefficient vector is publicly archived to support reproducibility. The resulting covariate balance, with absolute |SMD| below 0.10 for the large majority of covariates after stratification, is substantially better than what is achievable with manually specified propensity models in this clinical domain. The prespecified sensitivity analysis program, encompassing preference-score trimming, doubly adjusted modeling, three fixed administrative censoring horizons, and a dementia-only outcome restriction, was designed prior to examination of results and provides a transparent record of estimate stability across design perturbations. The concordance of all 6 analyses with the primary estimate supports the robustness of the finding. In addition, statistical power calculations confirmed adequate precision to detect HRs of approximately 1.15, and 271 negative-control outcomes were prespecified to evaluate residual systematic bias, although no postindex events were observed for any control outcome and empirical calibration could not be performed.

Finally, the complete analytic package, including ATLAS JSON cohort definitions, concept set files, *CohortMethod* R scripts, propensity model objects, and supporting code, is publicly archived and executable, enabling direct replication in any OMOP-standardized data network. This supports federated replication across OHDSI network sites and supports use of the present analysis as a reference for multi-database validation.

#### Limitations

A central limitation of this study is that exposure was defined entirely from clinical diagnosis codes, which capture clinically recognized HSV-1 disease rather than true biological infection status. Because HSV-1 establishes lifelong latency and reactivates without producing clinically attended symptoms in most infected individuals, only a small fraction of carriers ever receive a diagnostic code. Population-based serologic surveys estimate that approximately 65% of adults over 50 carry HSV-1 antibodies, yet clinical codes identify fewer than 5% of the seropositive population in EHR-based studies; the 6274 coded individuals in a data lake of 2.2 million patients are consistent with this gap. The practical consequence is that the comparison is not between infected and uninfected individuals, but between individuals with a coded HSV-1 diagnosis and a comparator group that, given the high background seroprevalence, almost certainly contains a large number of latently infected but never-coded individuals. This contamination of the unexposed group weakens the true exposure contrast and constitutes nondifferential exposure misclassification, which is expected to bias the HR toward the null. As discussed in the Comparison With Prior Work section, the observed HR is therefore attenuated and is best interpreted as a lower bound on the association between HSV-1 and cognitive impairment rather than as a direct estimate of the effect of infection itself. For the same reason, the coded-exposure design cannot separate the effect of HSV-1 infection from the effect of clinically apparent or recurrent HSV-1 disease, and the causal interpretation of this contrast should be made with corresponding caution. Restriction to HSV-1-specific *ICD* codes reduced, but did not eliminate, this misclassification; definitive resolution would require serologic or virologic confirmation of infection status in both cohorts, which was not available in this data source and is identified above as a priority for replication.

Beyond misclassification, a coded HSV-1 diagnosis may function in part as a marker of underlying host characteristics rather than as a direct causal exposure. Individuals who receive a clinical HSV-1 diagnosis are disproportionately those in whom the virus has reactivated with sufficient clinical severity to prompt medical evaluation, which is itself influenced by age-related immunosenescence, frailty, comorbidity burden, and relative immune dysregulation from other chronic conditions. Each of these features is independently associated with an elevated risk of cognitive impairment. Although the high-dimensional PS incorporated approximately 17,000 baseline covariates capturing medication use, condition history, clinical procedures, and health care usage, residual confounding by unmeasured dimensions of host susceptibility and immune status cannot be excluded. Accordingly, part of the observed association between coded HSV-1 and subsequent cognitive impairment may reflect shared antecedents of both outcomes rather than a direct effect of the virus on neurodegeneration. This consideration reinforces the need for replication in cohorts with richer immune, genetic, and frailty phenotyping before causal claims can be advanced.

Several additional limitations should be noted. Educational attainment and APOE genotype were not captured and could not be included in the PS model. Antiviral treatment patterns could not be systematically ascertained from diagnosis codes alone. Death and other competing outcomes were incompletely recorded, and the observed HR may therefore be subject to competing risks bias. The absence of postindex events for any of the 271 prespecified negative-control outcomes prevented empirical calibration; all reported estimates are uncalibrated, and residual systematic error cannot be formally quantified.

Preference-score distributions showed limited overlap between groups, with a c-statistic of approximately 0.92, indicating substantial baseline differences and supporting interpretation primarily within the region of covariate overlap. Although preference-score trimming yielded estimates consistent with the primary analysis, the comparator was encounter-anchored and defined by the absence of a recorded HSV-1 diagnosis rather than by a shared clinical indication. The high-dimensional PS improved balance across measured covariates, with absolute |SMD| below 0.10 for the large majority of covariates; however, residual confounding by unmeasured determinants of both HSV-1 diagnosis and cognitive impairment, including care-seeking behavior, symptomatic burden, frailty, and host susceptibility, cannot be excluded. The absolute risk difference curve narrows after approximately day 3000, primarily because of differential depletion of the HSV-1 at-risk pool rather than true convergence of cumulative incidence; tail-region estimates should be interpreted cautiously.

Eligibility criteria excluded individuals with recorded HIV, other selected neurotropic viral infections, or recent solid-organ or tissue transplantation in order to reduce confounding from established immunocompromising conditions. As a consequence, the findings may not generalize to immunocompromised populations, including people living with HIV, transplant recipients, and individuals on systemic immunosuppression, in whom HSV-1 reactivation frequency, clinical presentation, and central nervous system involvement are known to differ. Dedicated studies would be required before extrapolating these findings to such groups.

Finally, all data were drawn from a single Midwestern academic health system. Diagnostic coding intensity, patient demographics, and follow-up completeness may differ across institutions and regions. Replication in external OMOP-standardized networks with more diverse populations will be necessary to assess the generalizability of these findings.

### Conclusions

In a fifteen-year EHR cohort, patients with any HSV-1 diagnosis code experienced a 14% higher hazard of incident cognitive impairment, including dementia subtypes, than comparator patients with no recorded HSV-1 diagnosis (adjusted HR 1.14, 95% CI 1.03‐1.25). Absolute incidence differed by about four events per 1000 PY (Table 2), and PS-weighted survival curves diverged gradually, with separation emerging over extended follow-up (Figures 6–8). Although the estimated association is smaller than some earlier reports, it is consistent with estimates from recent longitudinal studies that applied extensive adjustment. Measurement limitations, incomplete death data, and potential residual confounding preclude causal inference; nevertheless, the consistent direction across sensitivity analyses supports a modest association within the observed data environment. A HR of 1.14 represents a modest effect on the individual level, and these findings alone do not support changes to current clinical practice for HSV-1 management or cognitive screening. Given the high prevalence of HSV-1, if the observed association is causal, even this modest relative excess could contribute meaningfully to population dementia burden. Future studies should integrate laboratory-confirmed HSV-1 typing, antiviral treatment histories, and genetic data to clarify whether virologic suppression is associated with reduced dementia incidence. If a causal relationship is confirmed, sustained antiviral suppression and future HSV-1 vaccines could become useful tools for reducing dementia incidence at the population level.

## Supplementary material

10.2196/83028 Multimedia Appendix 1 Analytic workflow diagram and cohort logic flow diagram.

10.2196/83028 Multimedia Appendix 2 HSV-1 cohort concept sets.

10.2196/83028 Multimedia Appendix 3 Covariate balance.

10.2196/83028 Multimedia Appendix 4 Non-HSV-1 cohort concept sets.

10.2196/83028 Multimedia Appendix 5 Incident cognitive impairment or dementia cohort concept sets.

10.2196/83028 Multimedia Appendix 6 Propensity model.

10.2196/83028 Multimedia Appendix 7 Negative control list.

10.2196/83028 Multimedia Appendix 8 Distribution of preference scores in exposure and comparator cohorts.

10.2196/83028 Checklist 1 STROBE checklist.

## Abbreviations

AD: Alzheimer disease
ADRD: Alzheimer disease and related dementias
APOE: apolipoprotein E.
CDM: Common data model
EHR: electronic health record
HIPAA: Health Insurance Portability and Accountability Act
HR: hazard ratio
HSV-1: herpes simplex virus type-1
*ICD-10-CM*: *International Classification of Diseases, Tenth Revision, Clinical Modification*
*ICD-9-CM*: *International Classification of Diseases, Ninth Revision, Clinical Modification*
OHDSI: Observational Health Data Sciences and Informatics
OMOP: Observational Medical Outcomes Partnership
PS: propensity score
PY: person-years
RR: relative risk
SMD: standardized mean difference
