# Supplementary material for: Association of Herpes Simplex Virus Type-1 With Dementia Outcomes: Longitudinal Retrospective Cohort Study Using Real-World Electronic Health Record Data
Source: JMIR Form Res. 2026 Sep 11;10:e83028. doi: 10.2196/83028 (PMC13568597; doi:10.2196/83028)
Supplement: Supplementary file 2 [file formative-v10-e83028-s008.docx]

Distribution of Preference Scores in Exposure and Comparator Cohorts

| Preference Score | Density (Target Group) | Density (Comparator Group) |
| --- | --- | --- |
| 0 | 0.043997 | 2.711278 |
| 0.010101 | 0.057115 | 3.924833 |
| 0.020202 | 0.07147 | 4.537198 |
| 0.030303 | 0.08659 | 4.475247 |
| 0.040404 | 0.101989 | 4.049881 |
| 0.050505 | 0.117303 | 3.521045 |
| 0.060606 | 0.132259 | 3.118904 |
| 0.070707 | 0.146687 | 2.835779 |
| 0.080808 | 0.160487 | 2.64393 |
| 0.090909 | 0.173613 | 2.495113 |
| 0.10101 | 0.186057 | 2.378467 |
| 0.111111 | 0.197842 | 2.293285 |
| 0.121212 | 0.208995 | 2.228239 |
| 0.131313 | 0.219544 | 2.180978 |
| 0.141414 | 0.22952 | 2.145608 |
| 0.151515 | 0.238957 | 2.120349 |
| 0.161616 | 0.247885 | 2.103078 |
| 0.171717 | 0.256329 | 2.090967 |
| 0.181818 | 0.264311 | 2.082 |
| 0.191919 | 0.271852 | 2.074677 |
| 0.20202 | 0.278969 | 2.068192 |
| 0.212121 | 0.285677 | 2.06207 |
| 0.222222 | 0.29199 | 2.055989 |
| 0.232323 | 0.297923 | 2.049739 |
| 0.242424 | 0.303487 | 2.043202 |
| 0.252525 | 0.308697 | 2.036316 |
| 0.262626 | 0.31357 | 2.029055 |
| 0.272727 | 0.31812 | 2.021413 |
| 0.282828 | 0.322361 | 2.013396 |
| 0.292929 | 0.326307 | 2.005014 |
| 0.30303 | 0.329972 | 1.996286 |
| 0.313131 | 0.333369 | 1.987236 |
| 0.323232 | 0.336512 | 1.977892 |
| 0.333333 | 0.339415 | 1.968285 |
| 0.343434 | 0.34209 | 1.958448 |
| 0.353535 | 0.344552 | 1.948416 |
| 0.363636 | 0.346813 | 1.938225 |
| 0.373737 | 0.348886 | 1.927909 |
| 0.383838 | 0.350783 | 1.917503 |
| 0.393939 | 0.352516 | 1.907041 |
| 0.40404 | 0.354098 | 1.896555 |
| 0.414141 | 0.355539 | 1.886077 |
| 0.424242 | 0.356852 | 1.875638 |
| 0.434343 | 0.358046 | 1.865267 |
| 0.444444 | 0.359133 | 1.854994 |
| 0.454545 | 0.360121 | 1.844846 |
| 0.464646 | 0.361021 | 1.83485 |
| 0.474747 | 0.361839 | 1.825034 |
| 0.484848 | 0.362583 | 1.815423 |
| 0.494949 | 0.363259 | 1.80604 |
| 0.505051 | 0.363872 | 1.79691 |
| 0.515152 | 0.364427 | 1.788054 |
| 0.525253 | 0.364926 | 1.779493 |
| 0.535354 | 0.365374 | 1.771248 |
| 0.545455 | 0.365772 | 1.763336 |
| 0.555556 | 0.366124 | 1.755775 |
| 0.565657 | 0.366432 | 1.748581 |
| 0.575758 | 0.366698 | 1.741768 |
| 0.585859 | 0.366925 | 1.735349 |
| 0.59596 | 0.367114 | 1.729335 |
| 0.606061 | 0.367267 | 1.723738 |
| 0.616162 | 0.367385 | 1.718566 |
| 0.626263 | 0.36747 | 1.713827 |
| 0.636364 | 0.367522 | 1.709527 |
| 0.646465 | 0.367542 | 1.705671 |
| 0.656566 | 0.367531 | 1.702261 |
| 0.666667 | 0.367489 | 1.699301 |
| 0.676768 | 0.367417 | 1.696789 |
| 0.686869 | 0.367316 | 1.694726 |
| 0.69697 | 0.367185 | 1.693112 |
| 0.707071 | 0.367025 | 1.691943 |
| 0.717172 | 0.366837 | 1.691216 |
| 0.727273 | 0.36662 | 1.690924 |
| 0.737374 | 0.366375 | 1.69106 |
| 0.747475 | 0.366101 | 1.691613 |
| 0.757576 | 0.365798 | 1.692572 |
| 0.767677 | 0.365466 | 1.693923 |
| 0.777778 | 0.365103 | 1.695649 |
| 0.787879 | 0.364709 | 1.697733 |
| 0.79798 | 0.364283 | 1.700156 |
| 0.808081 | 0.363824 | 1.702896 |
| 0.818182 | 0.363329 | 1.705929 |
| 0.828283 | 0.362797 | 1.709228 |
| 0.838384 | 0.362225 | 1.712766 |
| 0.848485 | 0.36161 | 1.716513 |
| 0.858586 | 0.360949 | 1.72044 |
| 0.868687 | 0.360236 | 1.724515 |
| 0.878788 | 0.359468 | 1.728704 |
| 0.888889 | 0.358639 | 1.732972 |
| 0.89899 | 0.357743 | 1.737284 |
| 0.909091 | 0.356772 | 1.741603 |
| 0.919192 | 0.355718 | 1.745891 |
| 0.929293 | 0.354569 | 1.750108 |
| 0.939394 | 0.353314 | 1.754215 |
| 0.949495 | 0.351939 | 1.758168 |
| 0.959596 | 0.350428 | 1.761922 |
| 0.969697 | 0.348765 | 1.765429 |
| 0.979798 | 0.346933 | 1.768641 |
| 0.989899 | 0.344915 | 1.771506 |
| 1 | 0.342689 | 1.773972 |

Table Note: The above Table displays the full distribution of preference scores for both the target (HSV-1 infected) and comparator (uninfected) cohorts, with each row indicating the score-specific density in each group. Preference scores, representing the estimated probability of group assignment given observed covariates, serve as a key diagnostic in assessing the adequacy of matching and overlap between exposure groups in propensity score analyses.
