# Supplementary material for: Association of Herpes Simplex Virus Type-1 With Dementia Outcomes: Longitudinal Retrospective Cohort Study Using Real-World Electronic Health Record Data
Source: JMIR Form Res. 2026 Sep 11;10:e83028. doi: 10.2196/83028 (PMC13568597; doi:10.2196/83028)
Supplement: Supplementary file 3 [file formative-v10-e83028-s009.docx]

**Checklist 1**

**STROBE Checklist**

| **Section/Topic** | **Item#** | **Recommendation** | **Page#** |
| --- | --- | --- | --- |
| Title and abstract | 1a | Indicate the study design with a commonly used term in the title or the abstract | 1 |
| Title and abstract | 1b | Provide in the abstract an informative and balanced summary of what was done and what was found | 1-2 |
| Introduction - Background/rationale | 2 | Explain the scientific background and rationale for the investigation being reported | 3 |
| Introduction - Objectives | 3 | State specific objectives, including any prespecified hypotheses | 3 |
| Methods - Study design | 4 | Present key elements of study design early in the paper | 4 |
| Methods - Setting | 5 | Describe the setting, locations, and relevant dates, including periods of recruitment, exposure, follow‑up, and data collection | 4-5 |
| Methods - Cohort definition | 6a | Give the eligibility criteria, and the sources and methods of selection of participants. Describe methods of follow‑up | 5-6 |
| Methods – Study design and Cohort definition | 6b | For matched studies, give matching criteria and number of exposed and unexposed (not applicable) | 4 |
| Methods – Cohort definition, target, comparator, and outcome | 7 | Clearly define all outcomes, exposures, predictors, potential confounders, and effect modifiers. Give diagnostic criteria, if applicable | 6 |
| Methods -PS estimation/measurement | 8 | For each variable of interest, give sources of data and details of methods of assessment (measurement). Describe comparability of assessment methods if there is more than one group | 4-6 |
| Methods - Bias, Negative control & calibration | 9 | Describe any efforts to address potential sources of bias | 6 |
| Methods - statistical power | 10 | Explain how the study size was arrived at | 8 |
| Methods - Quantitative variables | 11 | Explain how quantitative variables were handled in the analyses. If applicable, describe which groupings were chosen and why | 6 |
| Methods - Covariate | 12a | Describe all statistical methods, including those used to control for confounding | 6-7 |
| Methods - Statistical methods | 12b | Describe any methods used to examine subgroups and interactions | 10 |
| Methods - Statistical methods (covariate section) | 12c | Explain how missing data were addressed | 5 |
| Methods - Follow-up time | 12d | If applicable, explain how loss to follow‑up was addressed | 4 |
| Methods - Sensitivity analyses | 12e | Describe any sensitivity analyses | 13 |
| Results - Cohort accrual | 13a | Report numbers of individuals at each stage of study-e.g., numbers potentially eligible, examined for eligibility, confirmed eligible, included in the study, completing follow‑up, and analyzed | 8 |
| Results - Cohort accrual | 13b | Give reasons for non‑participation at each stage | 8 |
| Results - Cohort accrual | 13c | Consider use of a flow diagram | 8 (Figure 1) |
| Results - Descriptive data | 14a | Give characteristics of study participants (e.g., demographic, clinical, social) and information on exposures and potential confounders | 9-10 (Table 4) |
| Results - Descriptive data | 14b | Indicate number of participants with missing data for each variable of interest | 10 |
| Results - Follow-up time | 14c | Summaries follow‑up time (e.g., average and total amount) | 9 (Table 2) |
| Results - Outcome data | 15 | Report numbers of outcome events or summary measures over time | 9 (Table 3) |
| Results - Crude Incidence Rates of All Cause Cognitive Impairment | 16a | Give unadjusted estimates and, if applicable, confounder‑adjusted estimates and their precision (e.g., 95% confidence interval). Make clear which confounders were adjusted for and why they were included | 10 (Table 5) |
| Results - Main results | 16b | Report category boundaries when continuous variables were categorized | N/A |
| Results - Main results | 16c | If relevant, consider translating estimates of relative risk into absolute risk for a meaningful time period | 9 |
| Results - Other analyses | 17 | Report other analyses done—e.g., analyses of subgroups and interactions, and sensitivity analyses | 10-11 (Table 6) |
| Discussion - Key results | 18 | Summaries key results with reference to study objectives | 12 |
| Discussion - Limitations | 19 | Discuss limitations of the study, taking into account sources of potential bias or imprecision. Discuss both direction and magnitude of any potential bias | 15 |
| Discussion - Interpretation | 20 | Give a cautious overall interpretation of results considering objectives, limitations, multiplicity of analyses, results from similar studies, and other relevant evidence | 12-16 |
| Discussion - Generalizability | 21 | Discuss the generalizability (external validity) of the study results | 15-16 |
| Other information - Funding | 22 | Give the source of funding and the role of the funders for the present study and, if applicable, for the original study on which the present article is based | No funding sources |
