# Supplementary material for: Association of Herpes Simplex Virus Type-1 With Dementia Outcomes: Longitudinal Retrospective Cohort Study Using Real-World Electronic Health Record Data
Source: JMIR Form Res. 2026 Sep 11;10:e83028. doi: 10.2196/83028 (PMC13568597; doi:10.2196/83028)
Supplement: Supplementary file 4 [file formative-v10-e83028-s001.docx]

Figure A: Analytic Workflow Diagram

Figure B: Cohort Logic Flow Diagram
